# Comprehensive Computational Study of a Novel Chromene-Trione Derivative Bioagent: Integrated Molecular Docking, Dynamics, Topology, and Quantum Chemical Analysis

**DOI:** 10.3390/ijms26199661

**Published:** 2025-10-03

**Authors:** P. Sivaprakash, A. Viji, S. Krishnaveni, K. M. Kavya, Deokwoo Lee, Ikhyun Kim

**Affiliations:** 1 Department of Mechanical Engineering, Keimyung University, Daegu 42601, Republic of Korea; 2 Department of Physics, Kongunadu College of Engineering and Technology (Autonomous), Thottiyam, Tiruchirappalli 621215, India; 3 Department of Physics, University of Mysore, Mysore 570006, India; 4 Department of Computer Engineering, Keimyung University, Daegu 42601, Republic of Korea

**Keywords:** chromene, docking, dynamics, topological analysis, MEP

## Abstract

This work thoroughly investigated the compound 4-(2,5-Dimethoxyphenyl)-3,4-dihydrobenzo[g]chromene-2,5,10-trione (DMDCT) using molecular docking, quantum chemical analysis, and vibrational spectroscopy methodology. The medicinal chemistry group has been particularly interested in chromene and benzochromene derivatives due to their wide range of pharmacological actions, including anticancer, antibacterial, anti-inflammatory, antioxidant, antiviral, and neuroprotective capabilities. In this connection, DMDCT has been explored to evaluate its biological, electrical, and structural properties. DFT using the B3LYP functional and 6–31G basis was established to conduct theoretical computations with the Gaussian 09 program. The findings from these computations provide insight into the following topics: NBO interactions, optimal molecular geometry, Mulliken charge distribution, frontier molecular orbitals, and MEP. Second-order perturbation theory has been used to assess stabilization energies arising from donor–acceptor interactions. Furthermore, general features such as chemical hardness, softness, and electronegativity were studied. The results suggest that DMDCT has stable electronic configurations and biologically relevant active sites. This integrated experimental and theoretical study supports the potential of DMDCT as a practical scaffold for future therapeutic applications and contributes valuable information regarding its vibrational and electronic behavior.

## 1. Introduction

The continuous detection of novel bioactive molecules with enhanced pharmacological efficiency is now of primary importance in drug research and development. The natural and manufactured heterocyclic compounds have attracted greater attention owing to their extensive array of biological functions and medicinal uses [1]. In pharmaceutical chemistry, chromene and benzochromene derivatives are remarkable structures. Many compounds have been recognized for their pharmaceutical potential, including benzochromenes, which combine benzene and a chromene ring to enhance the drug-likeness and target binding [2]. Many synthetic and natural compounds have this main structure, confirming the wide range of biological activities. It has anti-inflammatory, hypolipidemic, antihypertensive, estrogenic, anticoagulant, analgesic, and antispasmodic activities [3,4,5,6]. Meanwhile, the benzochromene derivatives perform antiviral, antioxidant, central nervous, and antiplatelet aggregation system activities [7,8,9,10]. These compounds have been demonstrated to be antimicrobial [11,12,13,14], anti-tubercular [15,16], cardiovascular [17], vascular disrupting [18], antileishmanial [19,20], anti-rheumatic [21], neuroprotective (Alzheimer’s preventive) [22], anti-HIV [23], antiproliferative [24], anticancer [25], and anti-tumor [26,27]. The structural alterations in these compounds can appreciably involve their pharmacokinetic properties, receptor affinity, and metabolic stability, consequently influencing their therapeutic efficacy. In this study, DMDCT possesses critical pharmacophores, including methoxy-substituted phenyl rings and chromene structures. These features have been previously linked to enhanced biological activity and receptor specificity. Methoxy groups increase electron-donating actions, possibly enhancing interactions with biomolecular targets through π–π stacking and hydrogen bonding. Additionally, the dihydrobenzo[g]chromene-2,5,10-trione structure provides a stiff, planar configuration that facilitates advantageous docking contacts with enzymes or receptors associated with essential disease processes.

Molecular docking functions as a prediction instrument in structure-based drug design, facilitating the assessment of interactions between small compounds and target biomacromolecules, such as enzymes or receptors. It aids in evaluating the binding affinity, orientation, and conformational flexibility of ligands within the protein’s active site. In this work, molecular docking was used to evaluate the binding affinity of DMDCT with certain protein targets associated with cancer and neurological diseases. Docking simulations elucidate the binding mechanism and structural requirements of the ligand by analyzing hydrogen bonds, hydrophobic interactions, and electrostatic forces. MD simulations were achieved to evaluate the dynamic manners and permanence of the DMDCT–protein compound over time, complementing the docking experiments. Docking offers a static representation of interactions, whereas molecular dynamics simulations replicate authentic physiological conditions by including conformational flexibility, solvent interactions, and temperature variations. Key parameters, such as RMSD, Rg, and RMSF, are investigated to evaluate the overall stability and compactness of the ligand–receptor complex. The findings assist in corroborating the docking predictions and elucidating the dynamic properties of the recognition of molecules.

Quantum chemistry studies using DFT provide significant insights into the electrical and structural properties of molecules. This work employed DFT calculations using the B3LYP functional and the 6–31G basis set to enhance the geometry of DMDCT and identify several molecular descriptions. The optimized structural properties, which are attributed to bond lengths, bond angles, and dihedral angles, clarify the conformational preferences of the molecule and impact its binding efficiency. Electronic descriptors, such as HOMO and LUMO energies, are vital for understanding a molecule’s chemical reactivity and stability. The energy disparity between the HOMO and LUMO, referred to as the band gap, indicates the molecule’s kinetic stability and its potential for charge transfer interactions. A low energy gap indicates elevated chemical reactivity, making the molecule a suitable candidate for biological activity [28]. MEP maps were generated to depict the electron-rich and electron-deficient regions of the molecule, which are likely sites for interactions with nucleophiles and electrophiles in biological targets. The NBO analysis was employed to clarify intra- and inter-molecular charge transfer interactions, emphasizing critical donor–acceptor relationships and stabilization energies that affect the molecule’s electrical properties.

The DMDCT pharmacological potential can be assessed using vibrational spectroscopy, molecular docking, quantum chemistry, and RDG screening. The compound’s structural stability, binding affinity, electrical properties, and pharmacokinetics are detailed and explained in this study. These research findings may enable the systematic development of safer and more effective analogs or derivatives. Here, electron pair localization and chemical bonding in molecules are stimulated using the ELF and LOL analyses. Using these analytical techniques, one can more readily accept the properties of reactivity regions, high spatial resolution, and the nature of bonds and lone pairs.

Innovations in multifunctional chemicals with high bioavailability and target specificity are highly needed to combat global cancer and neurological illnesses. Hence, the chromene-based structure and advantageous functional group, DMDCT, is a good foundation for therapeutic research. In the present research, the findings were used to understand and develop computational chemistry and molecular biology for next-generation pharmaceutical applications.

## 2. Result and Discussion

### 2.1. Molecular Geometry

**Figure 1:**
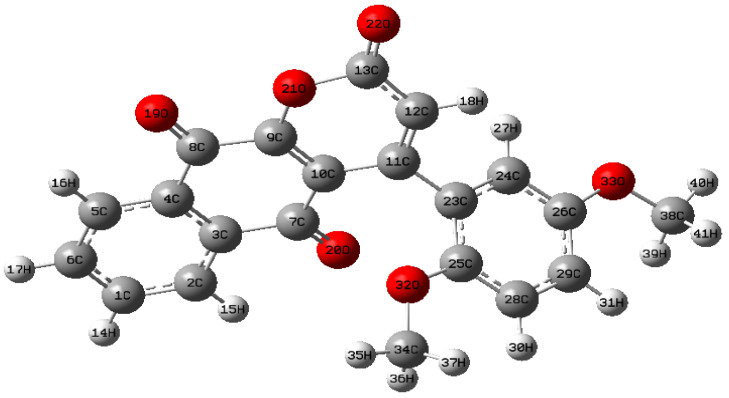
Molecular structure of DMDCT.

DMDCT’s molecular structure and atom numbering are shown in Figure 1. For a non-linear molecule of N atoms, the maximum number of potentially active fundamental vibrational modes is determined by the formula (3N − 6), which accounts for the exclusion of three translational and three rotational degrees of freedom. For DMDCT, including 41 atoms, the overall count of normal vibrational modes is 117 (i.e., 3 × 41 − 6 = 117). The vibrational modes are distributed throughout several symmetry species and are often categorized as in-plane and out-of-plane vibrations. In DMDCT, 117 normal modes include 38 in-plane vibrations and 38 out-of-plane vibrations, with the remaining modes associated with various stretching, bending, and torsional motions inside the molecule.

### 2.2. Structural Properties

DMDCT consists of five substituents: three carbonyl groups (C=O) and two methoxy groups (–OCH_3_). At the B3LYP/6–31G theoretical level, Gaussian 09 software calculated optimized geometric parameters, such as bond lengths, bond angles, and dihedral angles, as shown in Table 1. The molecule has 12 single C–C bonds, 7 C=C bonds, 3 C=O bonds, and 6 C-O bonds. The calculated C-C bond lengths vary from 1.36 to 1.43 Å. The longest C–C bond, measuring 1.43 Å, is found between C1-C6, C2-C3, C3-C4, C4-C5, C7-O20, C8-O19, C9-C10, C9-O21, C10-C11, C12-C13, C13-O22, C25-O32, C26-O33, O32-C34, and O33-C38. The bond lengths of C1-C2, C5-C6, and C13-O21 are equivalent, each measuring 1.36 Å. Similarly, C23-C25, C24-C26, C28-C29, C23-C25, and C24-C26 have an identical bond length of 1.39 Å. The C–O bond lengths vary from 1.36 Å to 1.432 Å, with the longest bond found in C13-O22 and the smallest in C13-O21, and the C–H bond lengths range from 1.07 Å to 1.43 Å, as determined at the B3LYP/6–31G level.

### 2.3. Vibrational Assignments

Table 2 presents the comprehensive vibrational assignments of the basic modes of DMDCT, including the measured and computed frequencies as well as normal mode descriptions. All computed vibrational frequencies were scaled using the standard B3LYP/6–31G scaling factor of 0.9 to correct for the systematic overestimation inherent in harmonic frequency calculations. This scaling ensures better agreement between the calculated and experimental FT-IR/FT-Raman spectra.

#### 2.3.1. C-H Vibration

The carbon–hydrogen stretching vibrations in the phenyl ring of DMDCT are detected within the range of 3082–2820 cm^−1^. Aromatic carbon–hydrogen stretching vibrations generally manifest in the infrared band between 3100 cm^−1^ and 3000 cm^−1^ [28], and the FT-IR bands detected at 3100, 3036, and 3030 cm^−1^ in this work are defined as aromatic carbon–hydrogen stretching modes [29]. The scaled vibrational frequencies, calculated using the B3LYP/6-31 + G(d,p) theoretical framework, demonstrate remarkable concordance with the experimental results. The in-plane bending vibrations of carbon–hydrogen bonds in aromatic rings often occur within the range of 1300–1000 cm^−1^. The Raman spectra of DMDCT reveal vibrations at 1282 cm^−1^, 1208 cm^−1^, and 1120 cm^−1^. Furthermore, for the DMDCT molecule, the C–H in-plane bending modes are computed to manifest at 1243 cm^−1^, 1223 cm^−1^, and 1216 cm^−1^. The carbon–hydrogen out-of-plane bending vibrations, which exhibit significant coupling with other modes, are generally detected within the range of 950–800 cm^−1^ [30]. In the present investigation, clearly identifies the modes at 948 cm^−1^, 933 cm^−1^, 932 cm^−1^, and 917 cm^−1^. These findings confirm the presence of characteristic aromatic ring vibrations in the DMDCT molecule and further validate the accuracy of the computational results.

#### 2.3.2. Carbon=Carbon, Carbon–Carbon, and Carbon–Carbon–Carbon Vibration

The stretching vibrations of carbon=carbon and carbon–carbon in aromatic rings often occur within the ranges of 1625 cm^−1^–1400 cm^−1^ and 1380 cm^−1^–1280 cm^−1^, respectively [31]. These vibrations are referred to as semicircle stretching vibrations due to the related ring deformation [32]. The ongoing examination of DMDCT identifies Carbon=Carbon stretching vibrations at 1626 cm^−1^ and 1565 cm^−1^ in the FT-IR spectra and at 1551 cm^−1^ and 1546 cm^−1^ in the FT-Raman spectrum. The C–C stretching vibration of the DMDCT molecule is seen at 1026 cm^−1^ in the FT-IR spectrum and at 994 cm^−1^ in the FT-Raman spectrum [33]. In a comparable system, Singh [34] previously documented the C–C–C stretching vibration at 1003 cm^−1^ (Raman) and 983 cm^−1^ (FT-IR). The C–C–C stretching mode for DMDCT is observed at 844 cm^−1^ in FT-IR and 840 cm^−1^ in FT-Raman in this study. The B3LYP/6–31G technique yielded a theoretical value that supports this observation. Additionally, the in-plane bending vibrations of the C–C–C group are seen at 796 cm^−1^, 774 cm^−1^, and 761 cm^−1^, whereas the out-of-plane bending vibrations are noted at 749 cm^−1^, 740 cm^−1^, and 718 cm^−1^. Theoretical models forecast a comparable bending mode at 695 cm^−1^.

#### 2.3.3. Methyl Group (CH_3_) Vibrations

In the vibrational analysis of substituted benzene rings, the existence of a methyl (–CH_3_) group results in nine distinctive fundamental modes, encompassing symmetric and asymmetric stretching, symmetric and asymmetric deformations, in-plane rocking (δ rock), in-plane bending (δ), out-of-plane rocking (γ rock), out-of-plane bending (γ), and twisting (ΓCH_3_) vibrations [35]. In the molecular structure of DMDCT, two methyl (CH_3_) groups are replaced on the aromatic ring system [36]. Typically, symmetric and asymmetric CH_3_ stretching vibrations are observed between the range of 2942 cm^−1^ and 2879 cm^−1^. N. Shanmugapriya et al. [37] report that CH_3_ asymmetric stretching vibrations are seen in FT-IR spectra at 2929 cm^−1^ (weak) and in the FT-Raman spectrum at 2947 cm^−1^ and 2933 cm^−1^. Sivakumar et al. [38] similarly identified pronounced peaks at 2922 cm^−1^, 2855 cm^−1^, and 2869 cm^−1^, which are equivalent to the symmetric and asymmetric CH_3_ stretching modes in both FTIR and FT-Raman spectra. Benzon [39] detected CH_3_ deformation vibrations at 1458, 1455, 1450, and 1435 cm^−1^, but Parveen et al. [40] attributed CH_3_ deformation to 1355 cm^−1^ in the IR spectra. In the current investigation, CH_3_ in-plane bending vibrations are designated at 1380 cm^−1^ and 1359 cm^−1^ in the FT-IR and FT-Raman spectra. The theoretical wavenumbers, computed by the B3LYP/6–31G approach, forecast the subsequent bending modes: δᵒᵖ = 695 cm^−1^, δⁱᵖᵇ = 589 cm^−1^, δˢᵇ = 528 cm^−1^, δᵒᵖʳ = 472 cm^−1^, and δⁱᵖʳ = 458 cm^−1^, corroborating the experimental results.

#### 2.3.4. C=O Vibration

The C=O stretching vibration of the carbonyl group is generally linked to a significant dipole moment and a pronounced stretching band, commonly detected in the spectral range of 1740–1660 cm^−1^, and the FT-IR spectra exhibited a moderate-intensity band at 1698/cm, attributable to the C=O stretching mode. An experimentally measured value exhibits a little divergence from the theoretically expected frequency of 1659 cm^−1^, determined using the B3LYP/6-311G computational method [41]. The in-plane bending vibrations of the carbonyl (C=O) group generally manifest within the 820–630 cm^−1^ range. Raajaraman et al. [42] documented the C=O in-plane bending vibration at 694 cm^−1,^ and the present study identifies a weak-intensity peak at 694 cm^−1^ in the FT-IR spectra, which corresponds to the C=O in-plane bending mode. The theoretical calculation employing the B3LYP/6–31G approach predicts this mode at roughly 695 cm^−1^, aligning well with the experimental result.

### 2.4. HOMO-LUMO Analysis

The HOMO–LUMO energy levels of the DMDCT molecule were determined via DFT with the B3LYP/6–31G basis set. The HOMO signifies the molecule’s potential to donate electrons, whereas LUMO denotes its ability to accept electrons. The FMO offers vital information regarding the compound’s chemical reactivity, kinetic stability, and electrical properties [43]. The HOMO–LUMO energy gap is a critical parameter for evaluating a molecule’s chemical stability, optical polarizability, and hardness–softness properties. A larger energy gap signifies a more rigid (less reactive) molecule, whereas a smaller gap denotes a more pliable (more reactive) character. The global hardness (*η*) of a molecule is determined by the formula [44]:

$$\eta \;=(-\varepsilon_{HOMO}+\varepsilon_{LUMO}/2)$$

**Figure 2:**
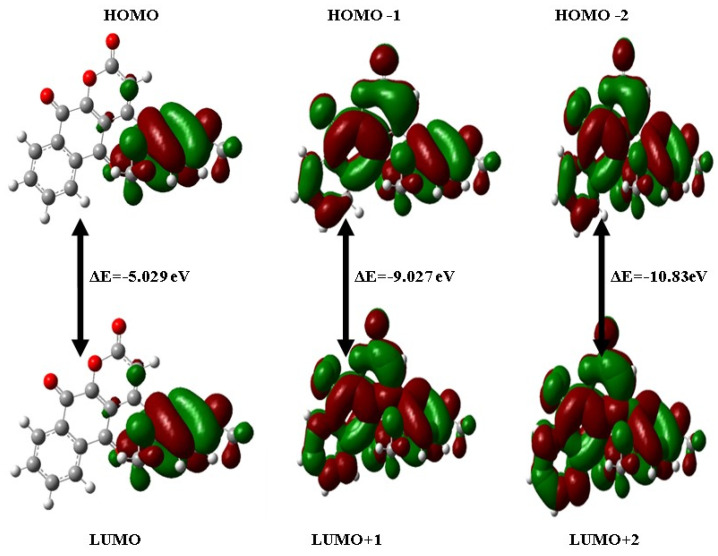
HOMO, HOMO-1, HOMO-2, LUMO, LUMO+1, and LUMO+2 pictures and their orbital energies of DMDCT; Positive wave function (Red), Negative wave function (Green).

This electronic spectrum transforms from the ground state to the first excited state, mostly due to a single electron excitation between the HOMO and LUMO orbitals. The HOMO energy is directly associated with the ionization potential, while the LUMO energy relates to the molecule’s electron affinity. Table 3 displays the calculated energies of the HOMO and LUMO, along with other orbitals such as HOMO-1, HOMO-2, LUMO+1, and LUMO+2. Figure 2 depicts the pertinent orbital plots and energy diagrams obtained using the B3LYP/6–31G method.

### 2.5. Global Chemical Reactivity Descriptors (GCRD)

We used the DFT method to look at the GCRD of the title compound. These include its global softness (σ), electronegativity (χ), global hardness (*η*), ionization potential (I), chemical potential (µ), electron affinity (*A*), and global electrophilicity index (*ω*), which were evaluated using density functional theory (DFT)-derived descriptors. Koopmans’ theory posits that the *E_HOMO* is directly correlated with the ionization potential (I), whereas the *E_LUMO* serves to approximate the electron affinity (*A*) [44].

I=−EHOMO

A=−ELUMO

µ=−(I+A)/2

η=(I−A)/2

σ=1/η

$$\omega ={µ}^{2}/2\eta$$

The chemical potential (µ), which signifies the propensity of electrons to depart from an equilibrium system, is a crucial parameter in elucidating molecular reactivity. These reactivity descriptors also provide insight into the molecule’s chemical stability, electrophilic/nucleophilic character, and reactive behavior. The FMO analysis was performed utilizing B3LYP with the 6–31G basis set, yielding valuable information on electronegativity, chemical hardness, and softness. The computed values of these descriptors are summarized in Table 3, supporting the understanding of the molecule’s potential applications in reactivity and biological interactions.

### 2.6. MEP Analysis

The MEP around a molecule represents its net electrostatic effect from its total charge distribution at that location. It correlates closely with molecular properties such as partial atomic charges, dipole moment, chemical reactivity, and electronegativity. The MEP analysis offers a visual approach to understanding the relative polarity of different regions within a molecule.

**Figure 3:**
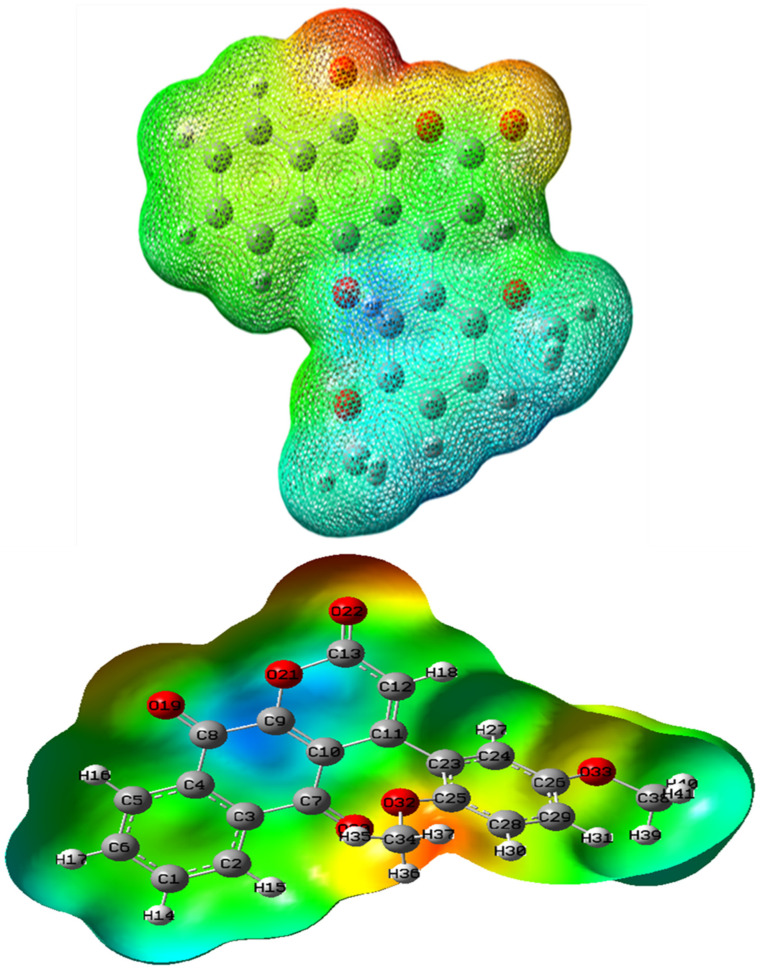
Molecular Electrostatic Potential of DMDCT.

In this study, an electron density iso-surface was generated and mapped with the electrostatic potential surface, highlighting possible reactive sites of the molecule. The electrostatic potential is shown using several colours: Red signifies areas with the lowest potential, whereas potential ascends in the sequence Red < Orange < Yellow < Green < Blue, with Blue denoting the highest potential zones. The DFT B3LYP technique with the 6–31G basis set in Gauss View [45] software (Gauss view 5.0) generated the compound DMDCT MEP surface. Figure 3 visualization aids in pinpointing prospective locations for electrophilic (negatively charged) and nucleophilic (positively charged) assaults. The red zones on the MEP surface are deemed strongly electronegative, promoting nucleophilic assault, whereas the blue portions are electron-deficient.

### 2.7. Natural Bond Orbital Analysis

In quantum chemistry, NBOs denote confined orbitals characterized by maximal electron density, providing a lucid representation of bonding interactions inside a molecule [46]. NBO analysis is an effective technique for investigating intramolecular charge transfer mechanisms, particularly the transference of electron density from occupied (donor) orbitals to empty (acceptor) orbitals. These interactions are essential for molecule stability, since they elucidate the mechanics of electron delocalization. The intensity of each donor–acceptor interaction is measured by the second-order stabilization energy, referred to as *E*(2), which is determined by second-order perturbation theory. This number indicates the extent of delocalization due to orbital overlap, enhancing comprehension of the molecule’s conjugation and stability.

$$E\left(2\right)=\Delta E_{ij}=q_{i}\frac{{\left(F_{i,j}\right)}^{2}}{\left(E_{j}-E_{i}\right)}$$

The *E*(2) value is influenced by parameters including the occupancy of the donor orbital, the diagonal components (ɛᵢ and ɛ*j*) representing the energies of the donor and acceptor orbitals, and the off-diagonal Fock matrix element, *F*(*i*,*j*), which characterizes the interaction between these orbitals [44]. An elevated *E*(2) value signifies a more robust donor–acceptor contact and a pronounced hyperconjugative effect, implying improved conjugation within the molecular structure. The interactions and their associated stabilization energies are presented in Table 4 and Table 5. The second-order perturbation analysis utilizing the NBO approach indicates considerable intramolecular hyperconjugative interactions, particularly with π-electrons, which greatly enhance the electronic stability of the molecule.

### 2.8. Mulliken Charge Calculation

**Figure 4:**
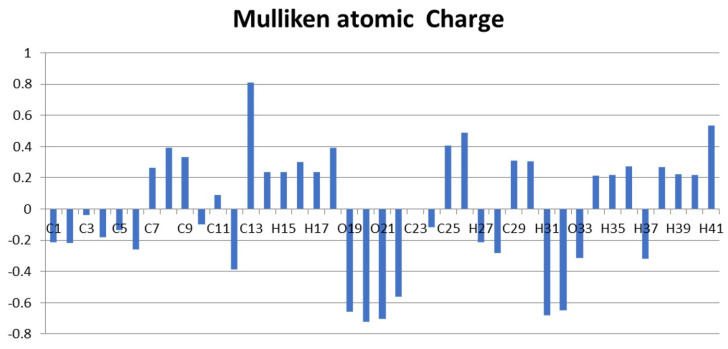
Mulliken atomic charges of DMDCT.

Mulliken atomic charge analysis is essential in quantum chemistry investigations of molecular systems, as it offers insights into charge distribution, which profoundly influences features such as dipole moment, molecular polarizability, and electronic structure. Table 6 and Figure 4 display the computed Mulliken atomic charges, derived from the electron population linked to the basic functions of each atom. The charge distribution in the specified molecule reveals that all hydrogen atoms, in addition to carbon atoms C7, C8, C9, C11, C13, C23, C25, and C26, exhibit positive charges. Conversely, the oxygen atoms and carbon atoms C1, C2, C3, C4, C5, C6, C10, C12, C24, H27, and C28 have a negative charge. C13, possessing a charge of +0.811, is among the most electropositive atoms, indicating a pronounced propensity to attract electrons. Conversely, the oxygen atoms O19 (−0.658) to O21 (−0.704) have considerable negative charges, signifying elevated electronegativity. Moreover, all hydrogen atoms bonded to the phenyl rings show electropositive characteristics, aligning with their anticipated chemical properties [47].

### 2.9. UV-Visible Spectra

**Figure 5:**
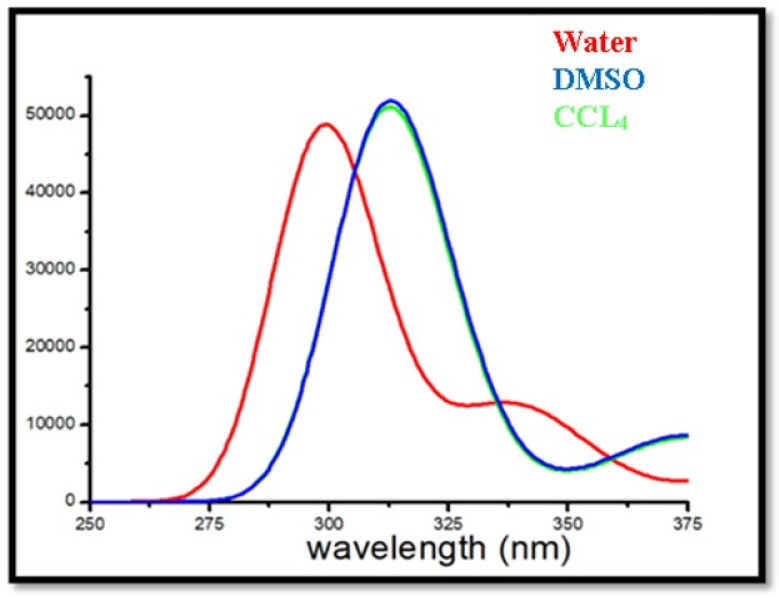
UV–Vis spectra of DMDCT in water, DMSO, and CCl_4_.

DFT analyses, employing the B3LYP/6–31G theoretical framework, were performed to evaluate several optical properties of the title compounds’ absorbance, oscillator strengths, excitation energies, and optimal geometry in the ground state. These simulations were essential for predicting the electronic absorption spectra of the molecule. Theoretical investigations have been conducted in many solvent environments, including carbon tetrachloride (CCl_4_), benzene, dimethyl sulfoxide (DMSO), and water, enabling a comparison of solvent effects on electronic transitions. In contrast to semi-empirical approaches for medium-sized molecules, DFT provides a dependable and computationally effective means of assessing electronic absorption spectra, as seen in Figure 5.

Table 7 summarizes the expected energy (*E*), absorption wavelengths, excitation, oscillator strengths (*f*), and the primary electronic transitions involved. The oscillator strength is a crucial parameter that signifies the probability of electronic transitions and may also be utilized to assess the LHE of the compound, which is vital for optoelectronic applications. The LHE may be computed using the following equation [48]:

LHE = 1 − 10^−f^

### 2.10. ELF and LOL Analysis

Topological investigations of the ELF and LOL are essential methodologies for examining the electrical structure of molecules. These approaches provide insights that are independent of the arbitrary choice of molecular orbitals and are directly based on the electron density distribution. Both ELF and LOL depict the electrical structure via localization domains (designated as η(r)) and attractors, which represent areas of covalent bonding, lone pairs, core electrons, and valence shells. The spatial arrangement of these attractors facilitates the categorization of core basins, often centered on atomic nuclei (except hydrogen), and valence basins, characterized by their connectedness to the core areas. This topological method offers a solid framework for defining chemical bonding based on electron localization.

**Figure 6:**
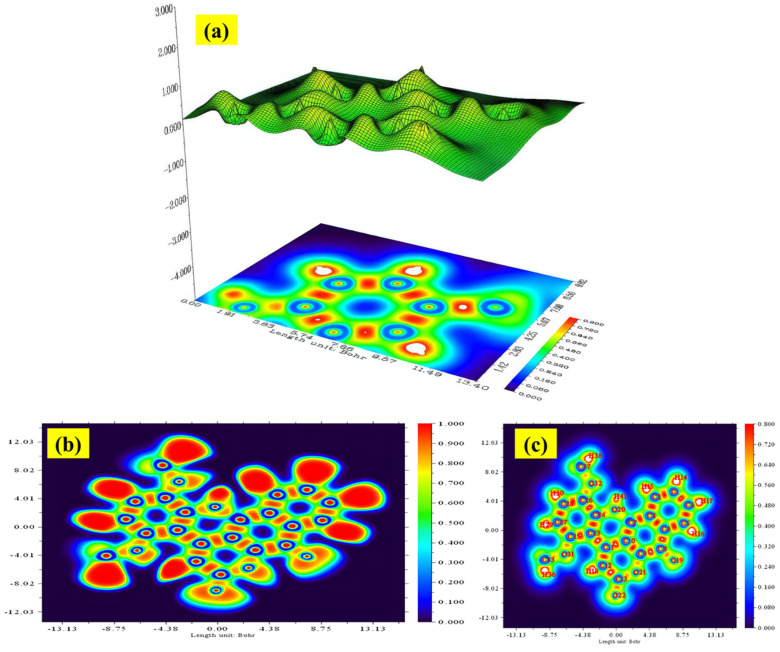
(**a**) ELF topology (**b**,**c**) LOL map of DMDCT in the region values of 1.0 to 0.0.

In ELF mapping, areas with minimal Pauli repulsion are depicted in blue, whereas areas of significant Pauli repulsion are illustrated in red. Regions with ELF < 0.5, signifying charge delocalization, are depicted in blue, whereas ELF > 0.5 areas—linked to covalent bonding—are represented in red. The ELF color gradient transitions from red (value about 1.0) to blue (value approximately 0.0). Figure 6a,b illustrates the electron density, ELF, and LOL plots for the chemical DMDCT. Figure 6c illustrates the LOL map, depicting the impact of electron localization with values spanning from 0.8 to 0.0. The areas of C–C and C–O covalent bonds display elevated LOL values, signifying substantial electron localization. Conversely, low localization zones manifest as blue, annular areas surrounding nuclei, particularly between valence and inner shells in heavier atoms. The illustrations also vividly illustrate the lone pairs on oxygen atoms.

### 2.11. RDG Analysis

The RDG function serves as a potent computational instrument for the visualization and analysis of non-covalent interactions (NCIs) in molecular structures [48]. It functions by assessing the real-space distribution of electron density, ρ(*r*), together with its first derivative. RDG aids in identifying and differentiating weak intermolecular interactions that influence molecular stability and reactivity.

$$RDG\left(r\right)=\frac{1}{2{\left(3\pi r^{2}\right)}^{1/2}}\frac{\left|\nabla \rho \left(r\right)\right|}{\rho {\left(r\right)}^{4/3}}$$

In *RDG* analysis, two-dimensional charts are created by graphing the *RDG* versus sign(*λ*_2_)*ρ*, where *λ*_2_ is the second eigenvalue of the electron density Hessian matrix. This product facilitates the categorization of interaction types: the sign of λ_2_ differentiates between attractive and repulsive interactions, and the size of *ρ*(*r*) signifies their intensity.

**Figure 7:**
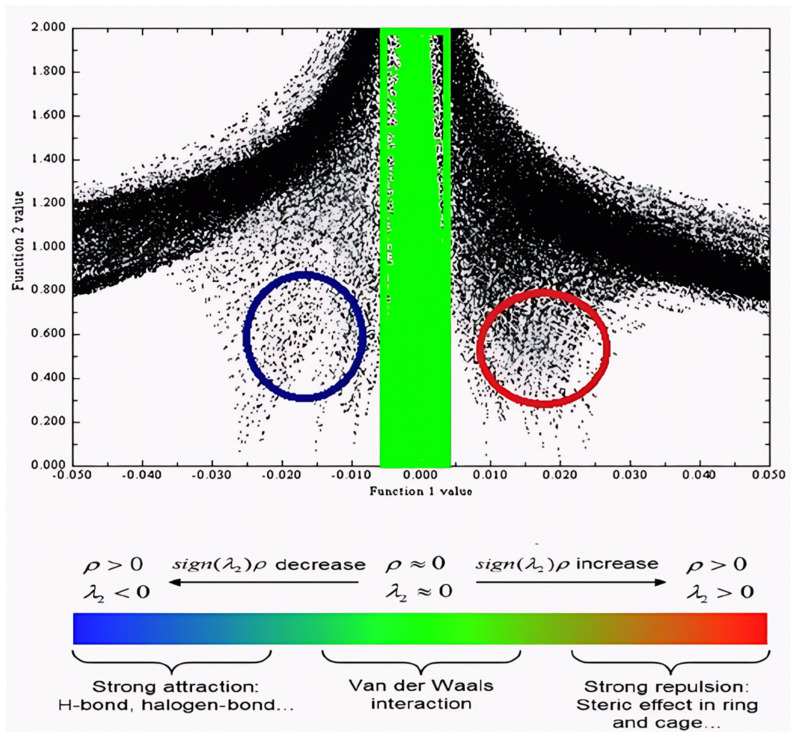
RDG map of DMDCT in the region values of 0.8 to 0.0 with atomic symbols.

The molecule DMDCT exhibits various non-covalent interactions as indicated by the RDG iso-surface and accompanying plot (Figure 7), based on sign(*λ*_2_)*ρ* values. Hydrogen bonding interactions are observable throughout the range of −0.050 to −0.015 a.u., characterized by prominent peaks in the negative area. Van der Waals interactions manifest within the range of −0.015 to 0.010 a.u., generally represented as wide and shallow characteristics. Steric repulsions, or exchange repulsion effects, range from 0.010 to 0.050 a.u., frequently appearing as peaks in the positive zone. In the RDG iso-surface maps, interaction types are color-coded for clarity: blue regions represent attractive interactions (e.g., hydrogen bonding), green regions identify weak dispersive forces (van der Waals), and red regions indicate repulsive interactions (steric effects). This graphical depiction provides a lucid and comprehensive comprehension of the molecule’s non-covalent interaction profile.

### 2.12. Molecular Docking Studies

MD simulations, collective with AI-powered drug design, have deeply progressed the competence of drug discovery by analyzing probable curative molecules with exactitude while reducing the need for wide lab experiments. These highly developed techniques enable the effective showing of vast compound libraries, calculation of binding strengths, and assessment of the immovability of drug–receptor composites at the molecular level. The integration of artificial intelligence enhances both the speed and dependability of these processes, and the creative drug development is further cost-effective and smoother than conventional approaches. By reducing protracted experimental measures and increasing early-stage recognition of valuable compounds, these computational strategies hasten innovation, improve protection, and lessen overall development costs.

**Figure 8:**
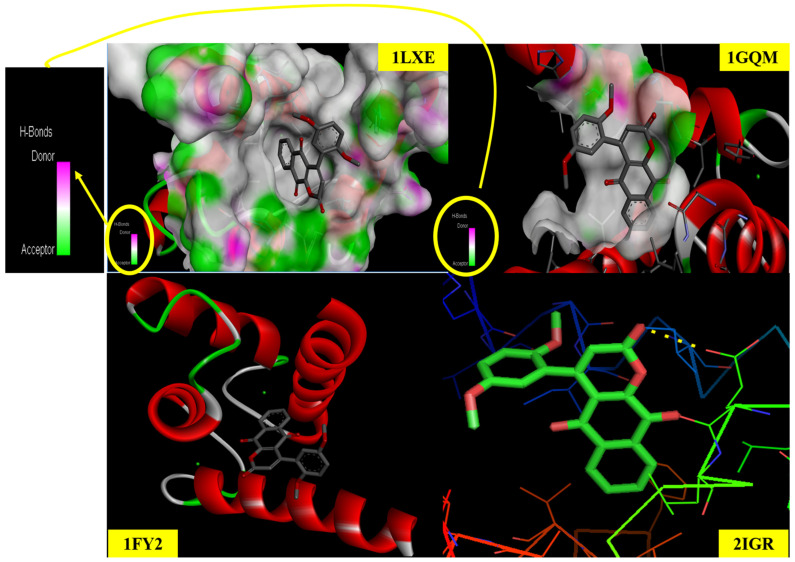
Molecular docking studies of protein–ligand interaction.

The ligand demonstrates significant binding affinities with many target proteins, as evidenced by its molecular docking interactions. Protein 1GQM engages with the ligand in its third conformer, demonstrating a minimum binding energy of −7.57 kcal/mol and an inhibition constant (Ki) of 2.82 µM. The protein 1FY2 demonstrates interaction through the sixth conformer of the ligand, with a binding energy of −7.11 kcal/mol and a Ki value of 6.17 µM. Similarly, protein 1LXE engages with the ligand in its first conformer, exhibiting a binding energy of −6.67 kcal/mol and an inhibition constant of 12.89 µM. The most robust interaction occurs with protein 2IGR, where the ligand’s second conformer has a minimum binding energy of −7.73 kcal/mol and an inhibition constant of 2.17 µM. Figure 8 depicts the ligand–protein binding interactions, while Table 8 and Table 9 summarize the associated binding energy values and inhibition constants.

### 2.13. MD Simulations

**Figure 9:**
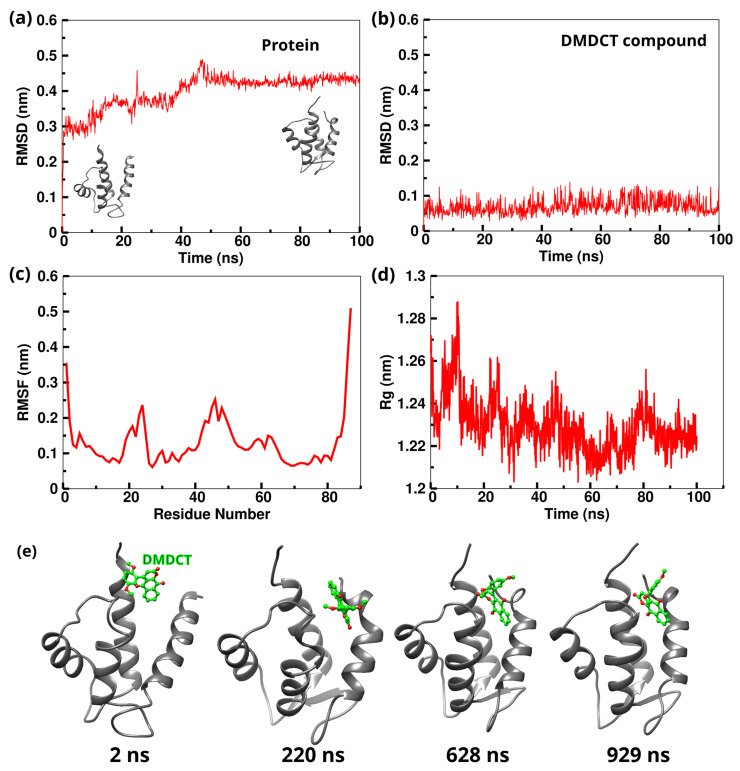
(**a**) RMSD graphs for the C-alpha atoms of the S100A12 protein showing the transition state. (**b**) RMSD graphs of the DMDCT compound in complex with the S100A12 protein. (**c**) RMSF plots for the S100A12 protein. (**d**) Radius of gyration for the S100A12 protein. (**e**) Conformational changes of the S100A12 protein–DMDCT complex at different stages of the molecular dynamics simulation.

**Figure 10:**
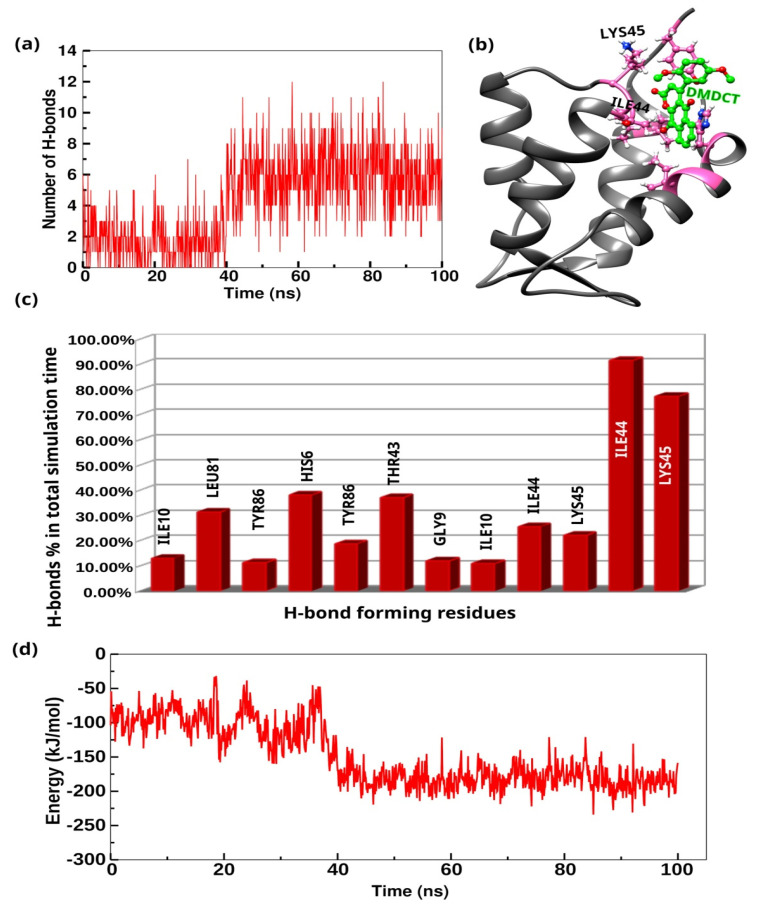
(**a**) The H-bond calculation between the S100A12 protein and the DMDCT compound over the simulation time. (**b**) Visual representation of the S100A12 protein in grey, with H-bond forming residues depicted in pink, and the DMDCT compound shown in green. (**c**) Bar graph showing the hydrogen bond occupancy, representing the percentage of the total simulation time that specific residues formed H-bonds with the DMDCT compound. (**d**) Non-bonded interaction energy (van der Waals and Coulombic).

MD simulations assessed the stability of the S100A12 protein complexed with the DMDCT compound. The 100 ns trajectories obtained from the MD simulations were analyzed for RMSD, RMSF, Rg, trajectory visualization, and hydrogen bonds (H-bonds). RMSD analysis revealed that the S100A12 protein remained stable in the presence of the DMDCT compound (Figure 9a). The RMSD of the 9s compound also indicated that it remained stable throughout the simulation time (Figure 9b). RMSF results highlighted fluctuations primarily in the loop regions of the protein (Figure 9c). The Rg analysis provided insights into the compactness of the simulated complexes, showing a decrease in the Rg value over the simulation time, which suggests that the complex became more compact (Figure 9d). Visualization of the trajectory frames revealed structural variations over time, indicating the structural transitions adopted by the protein under the influence of the DMDCT compound (Figure 9e). Further analysis of H-bond interactions between the protein and the DMDCT compound showed an increase in the number of H-bonds after 40 ns, indicating structural changes responsible for these increased interactions (Figure 10a). Notably, Lys45 (involved in 77.35% of the total simulation time) and Ile44 (involved in 91.42% of the total simulation time) were the primary residues forming H-bonds with the 9s compound for a significant portion of the simulation time (Figure 10b,c). These results collectively highlight the stability of the 9s compound in the protein binding pocket and its role in influencing the protein’s structural dynamics. In addition, non-bonded interaction energy calculations, including van der Waals and Coulombic contributions, demonstrated that the DMDCT compound exhibited a favorable interaction energy with the S100A12 protein (Figure 10d). These results collectively highlight the stability of the DMDCT compound in the protein binding pocket and its role in influencing the protein’s structural dynamics.

## 3. Experimental Method

Using a Perkin Elmer Spectrometer equipped with an MCT detector, a KBr beam splitter, and a “globar source” [Resolution of ±1 cm^−1^], the FTIR spectra of DMDCT were recorded from 4000 to 450 cm^−1^, at room temperature [model: Perkin Elmer, Waltham, MA, USA]. The Bruker instrument equipped with RFS27 Raman accessories measured the FT-Raman spectrum from 4000 to 0 cm^−1^ [Model: RFS27, Bruker, Billerica, MA, USA]. An Nd:YAG (1064 nm) with 200 mW power was used to excite the sample.

## 4. Computational Method

### 4.1. Density Functional Theory

The DFT calculations, including optimized bond lengths, dihedral angles, HOMO, LUMO, MEP, ESP, NBO, bond angles, UV, and Mulliken, were performed utilizing Becke’s B3LYP (3-parameter hybrid model and the Lee–Young–Parr correlation functional) approach via Gaussian 09 software [49]. The 6–31G basis set was used to ascertain the electronic molecular structure. To learn about the various locations where the molecule is attacked by electrophiles and nucleophiles, a molecular electrostatic potential diagram has been shown. As a result, the calculated frequencies, vibrational activity, polarization, MEP, hardness, softness, and bandgap were obtained. The Donor and Fock matrices in the NBO framework for the DMDCT molecule were computed using the B3LYP technique with a 6–31G basis set [45]. Stabilization energy was derived from second-order perturbation theory.

### 4.2. MD (Molecular Docking) Studies

MD analyses with AutoDock (AUTO DOCK 1.5.6) were performed to examine the binding interactions between the ligand and the target protein, DMDCT. The 3D structure of the target protein was acquired from the PDB and processed by eliminating water molecules and heteroatoms, then including polar hydrogens and Kollman charges. The ligand structure was subjected to energy minimization and subsequently translated to PDBQT format via AutoDock Tools [50]. The grid box specifications were set to include the protein’s active region, and the LGA was employed as the docking approach. Various docking conformations were produced, and the optimal posture was chosen based on binding energy and interaction patterns. The binding affinity (in kcal/mol), as well as hydrogen bonds, hydrophobic interactions, and π–π stacking, were evaluated to determine the stability and specificity of the protein–ligand complex. The results offer substantial insights into the potential of DMDCT as a therapeutic lead compound.

### 4.3. MD Simulations

The stability and finding affinity of the S100A12 protein MD complexed with the 9s were assessed using GROMACS 2023 software MD simulations [51,52,53,54,55,56]. The initial step involved separating the S100A12 protein and the DMDCT compound from the docked protein PDB file using the UCSF Chimera software (UCSF Chimera 1.18) [57]. Then, topology parameters for the S100A12 protein were assigned based on the CHARMM36 force field [58], while the topology information for the DMDCT compound was obtained from the CGenFF server [59]. The S100A12 protein bound to the DMDCT compound was then placed at the center of a triclinic box, ensuring a minimum distance of 1.2 nm between the protein and the box edges to avoid edge effects. The complex was solvated using the SPC water model [60], and Cl− and Na+ ions were added to neutralize the system’s net charge, and the decrease in energy minimization was executed to remove the steric effect. Following energy minimization, NVT equilibration was conducted at 300 K for 1 ns using the Berendsen thermostat [61]. Subsequently, NPT equilibration was carried out at 1 bar pressure for 1 ns, using the Parrinello–Rahman method for barostat pressure coupling [62]. Here, the PME method was used to deal with long-range electrostatic interactions [63], and H-bond constraints were enforced using the Linear Constraint Solver (LINCS) [64]. After equilibration, the systems were subjected to 100 ns of MD simulation.

The resulting trajectories were analyzed for RMSD, RMSF, and Rg using GROMACS in-built packages (Gromacs 2023). Additionally, H-bond analysis between the S100A12 protein and the DMDCT compound was conducted using VMD (Visual Molecular Dynamics) software (VMD 1.9.4) [65]. Visualization and image rendering were performed using VMD and Chimera packages. Graphs were drawn using Gnuplot (GNUPLOT 5.4).

### 4.4. Topological Investigations Studies

Topological investigation is crucial for inspecting the molecular structure and individuality of chemical compounds by centering on the connectivity and understanding of atoms and self-governing of their numerical form. This move is particularly precious in theoretical chemistry and pharmaceutical research, as it sheds light on a molecule’s immovability, reactivity, and e^-^ distribution. Through topological methods, scientists can expect biological functions, identify vital pharmacophores, and better realize structure–activity relationships (SAR). Key compensation of this technique embraces its short computational cost, high efficiency, and its capacity to improve and support experimental and quantum chemical findings, finally leading to the tactical development of new, effective, and safer drug candidates.

Analyses utilizing the RDG, LOL, and ELF were conducted with Multiwfn software (Multi wave 3.8 (dev)) to investigate the non-covalent interactions, electron localization, and bonding properties of DMDCT. The wavefunction file (.wfn or .fchk) produced by the DFT optimization using Gaussian 09 was utilized as input in Multiwfn [66]. RDG analysis was conducted to elucidate hydrogen bonds and weak intermolecular interactions, including van der Waals forces, utilizing scatter plots of RDG vs. sign(λ2)ρ to differentiate between attractive and repulsive interactions. The ELF and LOL functions were calculated to assess electron pair localization and delocalization within the molecular structure. ELF maps delineate areas of significant electron localization, including lone pairs and bonding zones, whereas LOL offers supplementary perspectives on the characteristics of orbital localization. The topological investigations provided a comprehensive knowledge of intra- and inter-molecular electron distribution, aiding in the interpretation of the molecule’s stability and reactivity.

## 5. Conclusions

The vibrational spectra of DMDCT were comprehensively examined in this study. Theoretical assumptions and empirically derived frequencies are in strong agreement. The FT-Raman and FTIR spectra of DMDCT were utilized to attribute all vibrational bands to various modes of vibration. The comprehensive vibrational assignments were evaluated according to PED. The optimal geometric dimensions and vibrational frequency assignments of the fundamental modes of the title compounds were ascertained by DFT/B3LYP/6–31G level calculations. The decreased values of the HOMO-LUMO energy gap indicate the biological activity of the compound, suggesting that these compounds exhibit significant nucleophilic reactivity. The NBO investigation indicates that the UV transitions’ maximum obtained wavelength is related to a number of probable intra- and intermolecular transitions. The tile molecule demonstrates non-covalent interactions, claims the RDG research. ELF and LOL analyses extensively describe the chemical structure, molecular bonding, and reactivity when applied for the quantitative analysis of aromaticity. The docking results demonstrated good to moderate antibacterial effectiveness against all tested pathogens when compared to a conventional drug. The chosen protein’s functional sites were docked with the ligand, and the lowest docking energy value was assessed. The planning of molecular dynamics simulation learn offers fundamental metrics such as RMSD, RMSF, and radius of gyration, revealing the consistent activity of the combinations throughout the simulation. In this study, we validated the stability of the DMDCT compounds within the S100A12 protein pocket through molecular dynamics simulations. The analysis of RMSD of DMDCT, hydrogen bond interactions, and electrostatic and van der Waals interactions between the DMDCT and the S100A12 protein confirmed that the DMDCT remains stable within the protein binding pocket through intermolecular interactions with the S100A12 protein. Notably, residues Ile44 and Lys45 of the S100A12 protein contributed significantly to hydrogen bond formation with the DMDCT compounds.

## Figures and Tables

**Table 1:** Optimized geometrical parameters of DMDCT obtained at B3LYP/6–31G basis sets.

Bond Length (A^0^):			
C1-C2	1.36	C13-O21	1.36
C1-C6	1.43	C13-O22	1.43
C1-H14	1.10	C23-C24	1.40
C2-C3	1.43	C23-C25	1.39
C2-15	1.10	C24-C26	1.39
C3-C4	1.43	C24-H27	1.10
C3-C7	1.40	C25-C28	1.40
C4-C5	1.43	C25-O32	1.43
C4-C8	1.40	C26-C29	1.40
C5-C6	1.36	C26-O33	1.43
C5-H16	1.10	C28-C29	1.39
C6-H17	1.10	C28-H30	1.10
C7-C10	1.40	C29-H31	1.10
C7-O20	1.43	O32-C34	1.43
C8-C9	1.40	O33-C38	1.43
C8-O19	1.43	C34-H35	1.07
C9-C10	1.43	C34-H36	1.07
C9-O21	1.43	C34-H37	1.07
C10-C11	1.43	C38-H39	1.07
C11-C12	1.36	C38-H40	1.07
C11-C23	1.54	C38-H41	1.07
C12-C13	1.43	C23-C25	1.39
C12-H18	1.10	C24-C26	1.39
**Bond Angle (^0^):**			
C2-C1-C6	120.4	C12-C13-O21	120.4
C2-C1-H14	121.0	C12-C13-O22	118.6
C6-C1-H14	118.6	C21-C13-O22	121.0
C1-C2-C3	120.8	C9-O21-C13	120.8
C1-C2-H15	121.2	C11-C23-C24	120.0
C3-C2-H15	118.1	C11-C23-C25	120.0
C2-C3-C4	118.8	C24-C23-C25	120.0
C2-C3-C7	121.7	C23-C24-C26	120.0
C4-C3-C7	119.5	C23-C24-H27	120.0
C3-C4-C5	118.8	C26-C24-H27	120.0
C3-C4-C8	119.5	C23-C25-C28	120.0
C5-C4-C8	121.7	C23-C25-O32	120.0
C4-C5-C6	120.8	C28-C25-O32	120.0
C4-C5-H16	118.1	C24-C26-C29	120.0
C6-C5-H16	121.2	C24-C26-O33	120.0
C1-C6-C5	120.4	C29-C26-O33	120.0
C1-C6-H17	118.6	C25-C28-C29	120.0
C5-C6-H17	121.0	C25-C28-H30	120.0
C3-C7-C10	121.0	C29-C28-H30	120.0
C3-C7-O20	119.5	C26-C29-C28	120.0
C10-C7-O20	119.5	C26-C29-H31	120.0
C4-C8-C9	121.0	C28-C29-H31	120.0
C4-C8-O19	119.5	C25-O32-C34	109.5
C9-C8-O19	119.5	C26-O33-C38	109.5
C8-C9-C10	119.5	O32-C34-H35	109.5
C8-C9-O21	121.7	O32-C34-H36	109.5
C10-C9-O21	118.8	O32-C34-H37	109.5
C7-C10-C9	119.5	H35-C34-H36	109.5
C7-C10-C11	121.7	H35-C34-H37	109.5
C9-C10-C11	118.8	H36-C34-H37	109.5
C10-C11-C12	120.8	O33-C38-H39	109.5
C10-C11-C23	119.6	O33-C38-H40	109.5
C12-C11-C23	119.6	O33-C38-H41	109.5
C11-C12-C13	120.4	H39-C38-H40	109.5
C11-C12-H18	121.0	H39-C38-H41	109.5
C13-C12-H18	118.6	H40-C38-H41	109.5
**Dihedral Angle (^0^):**			
C6-C1-C2-C3	0.16	C9-C10-C11-C23	−179.94
C6-C1-C2-H15	179.97	C10-C11-C12-C13	−0.1
H14-C1-C2-C3	−179.85	C10-C11-C12-H18	179.96
H14-C1-C2-H15	−0.04	C23-C11-C12-C13	179.9
C2-C1-C6-C5	0	C23-C11-C12-H18	−0.04
C2-C1-C6-H17	179.91	C10-C11-C23-C24	89.99
H14-C1-C6-C5	−179.98	C10-C11-C23-C25	−90.01
H14-C1-C6-H17	−0.08	C12-C11-C23-C24	−90.01
C1-C2-C3-C4	−0.25	C12-C11-C23-C25	89.99
C1-C2-C3-C7	179.76	C11-C12-C13-O21	0.08
H15-C2-C3-C4	179.93	C11-C12-C13-O22	180
H15-C2-C3-C7	−0.05	H18-C12-C13-O21	−179.98
C2-C3-C4-C5	0.18	H18-C12-C13-O22	−0.06
C2-C3-C4-C8	−179.92	C12-C13-O21-C9	−0.02
C7-C3-C4-C5	−179.83	O22-C13-O21-C9	−179.94
C7-C3-C4-C8	0.06	C11-C23-C24-C26	−179.97
C2-C3-C7-C10	−179.95	C11-C23-C24-H27	−0.05
C2-C3-C7-O20	−0.02	C25-C23-C24-C26	0.03
C4-C3-C7-C10	0.06	C25-C23-C24-H27	179.95
C4-C3-C7-O20	180	C11-C23-C25-C28	−179.98
C3-C4-C5-C6	−0.03	C11-C23-C25-O32	−0.01
C3-C4-C5-H16	−179.98	C24-C23-C25-C28	0.01
C8-C4-C5-C6	−179.92	C24-C23-C25-O32	179.99
C8-C4-C5-H16	0.13	C23-C24-C26-C29	−0.06
C3-C4-C8-C9	−0.1	C23-C24-C26-O33	179.96
C3-C4-C8-O19	180	H27-C24-C26-C29	−179.98
C5-C4-C8-C9	179.79	H27-C24-C26-O33	0.04
C5-C4-C8-O19	−0.11	C23-C25-C28-C29	−0.04
C4-C5-C6-C1	−0.07	C23-C25-C28-H30	179.98
C4-C5-C6-H17	−179.97	O32-C25-C28-H30	0
H16-C5-C6-C1	179.88	C23-C25-O32-C34	−155.32
H16-C5-C6-H17	−0.02	C28-C25-O32-C34	24.66
C3-C7-C10-C9	−0.15	C24-C26-C29-C28	0.03
C3-C7-C10-C11	179.9	C24-C26-C29-H31	−180
O20-C7-C10-C9	179.92	O33-C26-C29-C28	−179.98
O20-C7-C10-C11	−0.03	O33-C26-C29-H31	−0.02
C4-C8-C9-C10	0.01	C24-C26-O33-C38	140.96
C4-C8-C9-O21	−179.92	C29-C26-O33-C38	−39.02
O19-C8-C9-C10	179.91	C25-C28-C29-C26	0.01
O19-C8-C9-O21	−0.02	C25-C28-C29-H31	−179.96
C8-C9-C10-C7	0.12	H30-C28-C29-C26	−180
C8-C9-C10-C11	−179.94	H30-C28-C29-H31	0.03
O21-C9-C10-C7	−179.95	C25-O32-C34-H35	168.25
O21-C9-C10-C11	0	C25-O32-C34-H36	−71.75
C8-C9-O21-C13	179.91	C25-O32-C34-H37	48.25
C10-C9-O21-C13	−0.02	C26-O33-C38-H39	75.05
C7-C10-C11-C12	−179.99	C26-O33-C38-H40	−164.95
C7-C10-C11-C23	0.01	C26-O33-C38-H41	−44.95
C9-C10-C11-C12	0.07		

**Table 2:** Vibrational assignments of DMDCT calculated at B3LYP/6–31G set.

Species	Observed Frequency (cm^−1^)		Calculated Frequency (cm^−1^)	Vibrational Assignments
	Infrared	Raman	Scaled	
A		3076	3075	ν_CH_
A	3068		3067	ν_CH_
A	3044		3046	ν_CH_
			3022	ν_CH_
		2008	3011	ν_CH_
	3001		3002	ν_CH_
A			2985	ν_CH_
	2968		2971	ν_CH_
		2946	2945	ν_ass_CH_3_
	2928		2730	ν_ass_CH_3_
			2921	ν_ass_CH_3_
		2914	2915	ν_ass_CH_3_
			2867	ν_ass_CH_3_
	2835	2837	2836	ν_ass_CH_2_
	1795	1795	1798	ν_CO_
	1682	1682	1683	ν_CO_
			1675	ν_CO_
	1657	1659	1658	ν_CC_
	1642		1645	ν_CC_
			1619	ν_CC_
	1592	1594	1573	ν_CO_
			1575	ν_CO_
			1553	δ_CH_
			1522	δ_CH_
	1501		1500	ν_CO_
			1489	ν_CC_
			1478	ν_CO_
	1461	1459	1460	ν_CO_
			1452	ν_CO_
	1441		1440	δ_Opb_ CH_3_
			1427	δ_Opb_ CH_3_
	1419		1420	δ_CH_
			1402	δ_CH_
			1381	δ_CO_
	1364	1364	1365	δ_CO_
			1347	δ_CO_
	1331		1330	δ_ipb_ CH_3_
		1315	1316	δ_ipb_ CH_3_
	1303		1300	ν_CC_
	1275		1275	δ_CH_
		1267	1265	δ_CO_
			1238	δ_CO_
	1223		1225	ν_CC_
			1213	δ_CO_
	1206	1208	1206	δ_CO_
			1192	ν_CC_
	1183		1180	ν_CC_
			1171	ν_CC_
	1162	1165	1164	ν_CC_
			1143	ν_CC_
			1135	ν_CC_
	1125		1123	δ_sb_ CH_3_
			1063	δ_cb_ CH_3_
	1044		1042	δ_CH_
		1034	1033	δ_CH_
	1025		1025	γ_CO_
			1013	γ_CO_
			1001	γ_CO_
	985	987	986	δ_opr_ CH_3_
	951		950	γ_OO_
	936		937	γ_CO_
			925	δ CH_3_ CH_3_
	910		912	δ_CH_
		876	875	ν_CC_
			862	ν_CC_
	852		850	γ_CO_
		820	823	γ_CO_
			791	ν_CC_
	768		770	ν_CC_
	752	757	755	δ_ipr_ CH_3_
			747	δ_ipr_ CH_3_
			740	ν_CC_
			733	ν_CC_
	720		721	ν_CC_
	702		700	ν_CC_
			689	ν_CC_
	680	677	678	ν_CC_
			659	γ_CH_
			645	γ_CH_
			623	γ_CC_
	608		608	γ_CH_
	584		585	γ_CH_
	563		563	γ_CH_
	535		538	δ_ring_
			514	γ_CH_
	492		492	γ_CH_
	471		470	γ_CH_
		465	467	δ_ring_
			451	δ_ring_
	438		435	δ_ring_
			425	δ_ring_
			411	δ_ring_
			389	γ_CC_
			371	δ_ring_
			359	γ_ring_
			332	γ_ring_
			307	γ_ring_
			285	δ_ring_
			271	Butter fly
			253	Butter fly
			241	δ_ring_
			220	γ_ring_
			206	γ_ring_
			195	γ_ring_
			173	γ_ring_
			180	γ_ring_
			165	γ_ring_
			145	τ_ring_
			123	τ_ring_
			117	γ_ring_
			93	δ_ring_
			81	δ_ring_
			68	δ_ring_
			47	δ_ring_
			35	γ_ring_
			27	γ_ring_
			15	γ_ring_

**Table 3:** Comparison of HOMO and LUMO energy gaps and related molecular properties using B3LYP/6-31G).

Molecular	Energy	Energy Gap (eV)	*I* (eV)	*A* (eV)	*η* (eV)	χ (eV)	υ (eV)	µ (eV)	*ω* (eV)
Properties	(a.u)								
HOMO	−0.22286	−5.029	0.22286	0.03805	0.0924	0.13045	10.8225	−0.13045	0.092
LUMO	−0.03805								
LUMO + 2	0.08333								
HOMO − 1	−0.3058	−9.027	0.3058	0.02592	0.16586	0.13994	6.02918	−0.13994	0.059
LUMO + 1	0.02592								
HOMO − 2	−0.31473	−10.83	0.31473	−0.08333	0.19903	0.1157	5.0243	−0.1157	0.0336
LUMO + 2	0.08333								

**Table 4:** Natural band orbital analysis of DMDCT.

Band Orbital	Occupancies	EDA%	EDB%	Polarization Coefficient of Bond Orbital		Hybrid	S (%)	P (%)
				I Atom	II Atom			
C1-C2	1.98013–0.01490	49.68	50.32	0.7048	0.7094	SP^1.77^	36.15	63.85
C1-C6	1.97984–0.01723	50.47	49.53	0.7104	0.7038	SP^1.95^	33.92	66.08
C2-C3	1.97197–0.02273	47.62	52.38	0.6901	0.7238	SP^2.06^	32.66	67.34
C3-C4	1.95865–0.03097	51.51	48.49	0.7177	0.6964	SP^1.96^	33.81	66.19
C3-C7	1.96856–0.02442	49.93	50.07	0.7066	0.7076	SP^2.05^	32.83	67.17
C4-C5	1.97108–0.02281	52.32	47.68	0.7233	0.6905	SP^1.92^	34.27	65.73
C4-C8	1.97268–0.03450	52.45	47.55	0.7242	0.6895	SP^1.89^	34.59	65.41
C5-C6	1.98078–0.01461	49.77	50.23	0.7055	0.7088	SP^1.75^	36.34	63.66
C7-C10	1.96895–0.02785	48.23	51.77	0.6945	0.7195	SP^1.62^	38.17	61.83
C7-O20	1.98895–0.02565	27.03	72.97	0.5199	0.8542	SP^3.86^	20.57	79.43
C8-C9	1.98041–0.03206	48.01	51.99	0.6929	0.7211	SP^1.81^	35.55	64.45
C8-O19	1.98591–0.00898	43.79	56.21	0.6617	0.7497	SP^2.71^	26.97	73.03
C9-C10	1.96499–0.03228	46.38	53.62	0.6810	0.7323	SP^1.86^	34.99	65.01
C9-021	1.98588–0.02521	31.63	68.37	0.5624	0.8269	SP^3.20^	23.78	76.22
C10-C11	1.97023–0.02796	49.68	50.32	0.7048	0.7094	SP^2.18^	31.46	68.54
C11-C12	1.97403–0.02274	52.42	47.58	0.7240	0.6898	SP^1.61^	38.33	61.67
C11-C23	1.96904–0.02814	47.02	52.98	0.6857	0.7278	SP^2.56^	28.09	71.91
C12-C13	1.98424–0.03379	52.65	47.35	0.7256	0.6881	SP^2.11^	32.20	67.80
C13-O21	1.99265–0.05963	30.56	69.44	0.5528	0.8333	SP^2.67^	27.22	72.78
C13-O22	1.99096–0.01979	41.87	58.13	0.6470	0.7625	SP^2.14^	31.88	68.12
O20-C24	1.86519–0.04415	64.32	35.68	0.8020	0.5973	SP^2.88^	25.74	74.26
C23-C24	1.96447–0.02870	48.98	51.02	0.6999	0.7142	SP^1.98^	33.51	66.49
C23-C25	1.96957–0.02476	51.39	48.61	0.7169	0.6972	SP^1.85^	35.11	64.89
C24-C26	1.96617–0.02731	52.17	47.83	0.7223	0.6916	SP^1.50^	39.97	60.03
C25-C27	1.97902–0.02202	49.94	50.06	0.7067	0.7075	SP^1.65^	37.68	62.32
C25-O31	1.99016–0.02311	34.90	65.10	0.5908	0.8068	SP^3.08^	24.52	75.48
C26-C28	1.97444–0.02088	50.56	49.44	0.7110	0.7032	SP^1.57^	38.96	61.04
C26-O32	1.98985–0.02061	35.74	64.26	0.5978	0.8016	SP^3.11^	24.34	75.66
C27-C28	1.97308–0.01800	49.99	50.01	0.7071	0.7071	SP^1.83^	35.29	64.71
O31-C33	1.99357–0.00515	69.23	30.77	0.8320	0.5547	SP^2.53^	28.30	71.70
O32-C37	1.99361–0.00375	69.28	30.72	0.8323	0.5543	SP^2.44^	29.07	70.93

**Table 5:** The Second-order perturbation energies *E*(2) (kcal/mol) corresponding to the most important charge transfer interactions (donor–acceptor) of DMDCT.

Donor NBO (*i*)	ED(*i*) e	Acceptor NBO (*j*)	ED (*j*) e	*E*(2) kcal/mol	*E*(*j*)-*E*(*i*) a.u.	*F*(*i*,*j*) a.u.
σ C1-C2	1.98013	σ* C2-C3	0.95499	5.02	1.74	0.083
π C1-C2	1.79066	π* C3-C7	0.33282	36.68	0.44	0.126
σ C1-C6	1.97984	σ* C1-C2	0.88828	4.42	1.79	0.079
σ C1-H14	1.98487	σ* C2-C3	0.69559	4.75	1.48	0.075
σ C2-C3	1.97197	σ* C3-C4	0.89351	5.91	1.7	0.09
σ C2-H15	1.98440	σ* C1-C6	0.69421	3.93	1.49	0.068
σ C3-C4	1.95865	σ* C3-C7	0.89084	5.77	1.71	0.089
π C3-C7	1.68109	π* C9-C10	0.35425	55.66	0.47	0.15
σ C4-C5	1.97108	σ* C4-C8	0.87139	6.63	1.74	0.096
σ C4-C8	1.97268	σ* C4-C5	0.92384	6.44	1.73	0.094
π C5-C6	1.75574	LPσ C4	0.31189	53.37	0.24	0.133
σ C5-H16	1.98143	σ* C1-C6	0.66643	4.74	1.47	0.074
σ C6-H17	1.98483	σ* C1-C2	0.68152	3.16	1.58	0.063
σ C7-C10	1.96895	σ* C3-C7	0.98067	8	1.8	0.107
σ C7-O20	1.98895	σ* C9-C10	1.23056	1.54	2	0.05
σ C8-C9	1.98041	σ* C9-C10	0.94869	5.46	1.72	0.087
σ C8-O19	1.98591	σ* C3-C4	0.88766	3.52	1.7	0.069
π C9-C10	1.71142	LPσ* C8	0.34716	67.39	0.34	0.159
σ C9-O21	1.98588	σ* C7-C10	1.09962	1.86	1.9	0.053
σ C10-C11	1.97023	σ* C11-C12	0.95750	6.86	1.8	0.1
σ C11-C12	1.97403	σ* C10-C11	1.01857	7.97	1.75	0.106
π C11-C12	1.81365	π* C13-O22	0.39575	35.58	0.49	0.121
σ C11-C23	1.96904	σ* C23-C25	0.90300	3.73	1.64	0.07
σ C12-C13	1.98424	σ* C11-C23	0.97728	5.62	1.53	0.083
σ C12-H18	1.97133	σ* C10-C11	0.72813	5.77	1.46	0.082
σ C13-O21	1.99265	σ* C8-C9	1.19856	2.03	2.05	0.058
σ C13-O22	1.99096	σ* C11-C12	0.97399	2.69	1.82	0.063
π C13-O22	1.96408	π* C11-C12	0.40948	12.12	0.56	0.076
σ C23-C24	1.96447	σ* C24-C26	1.06814	6.64	1.78	0.097
π C23-C24	1.62979	π* C26-C28	0.47461	39.47	0.53	0.13
σ C23-C25	1.96957	σ* C25-C27	1.06092	5.79	1.78	0.091
σ C24-C26	1.96617	σ* C23-C24	1.09459	8.02	1.8	0.107
σ C25-C27	1.97902	σ* C23-C25	1.05789	6.52	1.8	0.097
π C25-C27	1.60918	π* C23-C24	0.41544	66.65	0.42	0.156
σ C25-O31	1.99016	σ* C23-C24	1.13212	2.04	1.84	0.055
σ C26-C28	1.97444	σ* O20-C24	1.06436	7.09	1.37	0.088
π C26-C28	1.66248	π* C25-C27	0.43109	47.22	0.49	0.136
σ C26-O32	1.98985	σ* C23-C24	1.14113	1.91	1.85	0.053
σ C27-C28	1.97308	σ* C25-O31	1.04167	4.59	1.46	0.073
σ C27-H29	1.97890	σ* C23-C25	0.81087	4	1.55	0.07
σ C28-H30	1.97669	σ* C24-C26	0.80388	4.35	1.51	0.072
σ O31-C33	1.99357	σ* C23-C25	1.11124	3.97	1.85	0.077
σ O32-C37	1.99361	σ* C24-C26	1.12540	3.9	1.83	0.076
σ C33-H34	1.99466	σ* C27-H29	0.79099	0.86	1.39	0.031
σ C33-H35	1.99469	σ* C27-H29	0.79059	0.82	1.39	0.03
σ C33-H36	1.99233	σ* C25-O31	0.77809	3.13	1.2	0.055
σ C37-H38	1.99227	σ* C26-O32	0.78510	3.23	1.19	0.055
σ C37-H39	1.99493	σ* C37-H38	0.79634	0.81	1.49	0.031
σ C37-H40	1.99490	σ* C28-H30	0.79738	0.81	1.39	0.03
LPσ O19	1.98865	σ* C4-C8	0.97085	2.15	1.84	0.056
LPσ O20	1.96990	σ* C23-C24	0.88670	5.57	1.6	0.084
LPσ O31	1.95396	σ* C12-H18	0.88864	17.58	1.59	0.15
LPπ O21	1.76389	π* C13-O22	0.45550	83.46	0.55	0.194
LPπ O32	1.87472	π* C26-C28	0.54998	39.31	0.6	0.144
LPπ O31	1.89710	π* C25-C27	0.54984	29.88	0.61	0.128
π* C1-C2	0.01490	π* C5-C6	0.89676	150.22	0.02	0.108
π* C23-C24	0.02870	π* C25-C27	0.70832	174.07	0.06	0.135
π* C26-C28	0.02088	π* C25-C27	0.71241	536.32	0.01	0.116
LP(3) O19	1.63920	LPσ* C8	0.21450	211.21	0.21	0.214

**Table 6:** Mulliken charge calculation of DMDCT.

Atom	Mulliken Atomic Charge	Atom	Mulliken Atomic Charge
C1	−0.211	O22	−0.561
C2	−0.216	C23	0.001
C3	−0.040	C24	−0.118
C4	−0.183	C25	0.406
C5	−0.137	C26	0.490
C6	−0.259	H27	−0.213
C7	0.262	C28	−0.283
C8	0.393	C29	0.310
C9	0.334	H30	0.304
C10	−0.099	H31	−0.679
C11	0.089	O32	−0.648
C12	−0.389	O33	−0.314
C13	0.811	C34	0.215
H14	0.236	H35	0.218
H15	0.237	H36	0.272
H16	0.302	H37	−0.320
H17	0.235	C38	0.268
H18	0.392	H39	0.225
O19	−0.658	H40	0.219
O20	−0.723	H41	0.536
O21	−0.704		

**Table 7:** Light harvesting efficiency (LHE) of DMDCT.

Derivative	Energy (eV)	Wave Length (nm)	Osc. Strength	Symmetry	Major Contributions	Minor Contribution	LHE
benazene	2.296	539	0.0971	Singlet-sym	HOMO-LUMO (90%)	H-1-LUMO (3%)	0.2003
	3.307	374	0.1186	Singlet-sym	HOMO-L + 1 (78%)	HOMO-L + 2 (8%)	0.2389
	3.965	312	0.1162	Singlet-sym	H-1-LUMO (56%)	H-1-L+1 (11%)	0.2347
CCl_4_	2.285	542	0.0961	Singlet-sym	HOMO-LUMO (91%)	HOMO-L + 2 (8%)	0.1985
	3.304	375	0.1148	Singlet-sym	HOMO-L + 1 (78%)	H-1-L+1 (10%)	0.2322
	3.968	312	0.706	Singlet-sym	H-1-LUMO (56%)	H-4-LUMO (14%)	0.8032
DMSO	3.046	406	0.0906	Singlet-sym	HOMO-LUMO (90%)	H-5-LUMO (4%)	0.1882
	3.659	338	0.17	Singlet-sym	HOMO-L + 1 (80%)	H-5-LUMO (3%)	0.3239
	4.147	298	0.6729	Singlet-sym	H-1-LUMO (47%)	HOMO-L + 2 (4%)	0.7876
Ethonal	3.0100	411	0.0864	Singlet-sym	HOMO-LUMO (90%)	H-5-LUMO (4%)	0.1804
	3.644	340	0.1596	Singlet-sym	HOMO-L + 1 (80%)	H-1-LUMO (2%)	0.3075
	4.152	298	0.6668	Singlet-sym	H-4-LUMO (30%)	H-1-L+1 (8%)	0.7846
Methonal	3.0305	409	0.0855	Singlet-sym	HOMO-LUMO (90%)	H-5-LUMO (3%)	0.1787
	3.657	339	0.1568	Singlet-sym	HOMO-L + 1 (80%)	H-1LUMO (2%)	0.3030
	4.162	297	0.683	Singlet-sym	H-1-LUMO (46%)	H-2-LUMO (2%)	0.7925
Water	3.067	404	0.0874	Singlet-sym	HOMO-LUMO (90%)	H-5-LUMO (4%)	0.1822
	3.676	337	0.1601	Singlet-sym	HOMO-L + 1 (80%)	H-5-LUMO (3%)	0.3083
	4.165	297	0.6554	Singlet-sym	H-1-LUMO (46%)	HOMO-L + 2 (4%)	0.7788 HO

**Table 8:** Binding energy and inhibition constant of ligands DMDCT with some proteins.

Ligand	Conformer	1gqm		1FY2		1lxe		2IGR	
		B.E (Kcal/mol)	Inhibition Const. (µm)	B.E (Kcal/mol)	Inhibition Const. (µm)	B.E (Kcal/mol)	Inhibition Const. (µm)	B.E (Kcal/mol)	Inhibition Const. (µm)
DMCT	1	−7.32	4.28	−6.93	8.35	−6.67	12.89	−7.00	7.45
	2	−7.27	4.70	−6.01	37.5	−5.15	167.89	−7.73	2.17
	3	−7.57	2.82	−5.90	46.96	−5.27	144.24	−6.99	7.49
	4	−7.27	4.67	−7.08	6.43	−5.96	43.12	−7.58	2.77
	5	−7.09	6.34	−6.37	21.33	−5.89	48.18	−6.99	4.49
	6	−6.71	12.02	−7.11	6.17	−5.21	152.38	−6.99	7.52
	7	−7.36	4.01	−6.27	25.40	−5.69	67.61	−6.96	7.96
	8	−7.29	4.55	−6.55	15.89	−5.44	102.44	−7.38	3.83
	9	−7.14	5.83	−5.46	99.05	−5.23	147.40	−7.13	5.98
	10	−7.33	4.22	−7.03	7.03	−5.48	96.67	−6.99	7.53

**Table 9:** Band distance ligand and residues.

9 Ligand	Protein PDB ID (1fy2)		Protein PDB ID (1xe)		Protein PDB ID (2igr)		Protein PDB ID (1gqm)	
	Residues	Bond Distance	Residues	Bond Distance	Residues	Bond Distance	Residues	Bond Distance
DMCT	A:ILE:170	5.07	A:GLV:82	3.94	TRP A: 12	2.54	ALA H: 78	3.93
	A:GLY:173	1.98	A:THR:83	2.69	LYS A: 16	5.38	ALA J: 62	3.43
	A:HIS:24	3.20	A:VAL:80	5.43	LYS A: 26	2.03	LYS H: 82	3.06
	A:GLN:175	3.07	A:LYS:81	1.99	TRP A: 25	5.07	ILE H: 79	2.91
	A:ASN:172	2.21	A:LYS:105	2.05	LYS A: 26	3.94	ALA H: 78	4.90
Estimated inhibition const (µm)	6.17		12.89		2.17		2.82	
Binding Energy	−7.11		−6.67		−7.73		−7.57	

## Data Availability

Data will be made available on request.

## Abbreviations

Rg	Radius of Gyration
MD	Molecular dynamics
PDB	Protein Data Bank
HOMO	Highest Occupied Molecular Orbital
MEP	Molecular Electrostatic Potential
LUMO	Energy of the lowest unoccupied molecular orbital
LHE	Light harvesting efficiency
*E_LUMO*	Lowest Unoccupied Molecular Orbital
PED	potential energy distribution
SPC	Simple Point Charge
DFT	Density Functional Theory
LOL	Localized Orbital Locator
RMSF	Root-mean-square fluctuation
RMSD	Root-mean-square deviation
LGA	Lamarckian Genetic Algorithm
ELF	Electron Localization Function
NBO	Natural Bond Orbital
*E_HOMO*	Energy of the highest unoccupied molecular orbital
FMO	Frontier molecular orbital
PME	Particle Mesh Ewald

## References

[1] da Silva A.J., Buarque C.D., Brito F.V., Aurelian L., Macedo L.F., Malkas L.H., Hickey R.J., Lopes D.V., Noël F., Murakami Y.L. (2002). Synthesis and preliminary pharmacolgical evaluation of new (±) 1,4-naphthoquinones structurally related to lapachol. Bioorganic Med. Chem..

[2] Mofakham H., Ghadari R., Shaabani A., Pedarpour M., Ghasemi S. (2013). “On-water” organic synthesis: L-proline catalyzed synthesis of pyrimidine-2,4-dione-, benzo[*g*]- and dihydropyrano [2,3-*g*]chromene derivatives in aqueous media. J. Iran. Chem. Soc..

[3] Verma V.S., Badwaik H.R., Vaishnav Y., Alexander A. (2022). Synthesis, characterization, molecular modelling and biological evaluation of substituted benzo (h) Chromene-3-carboxylate derivatives as a potential agent for the treatment of hyperlipidemia. Indian J. Pharm. Sci..

[4] Kajal C., Prima F. (2021). Callypyrones from marine Callyspongiidae sponge Callyspongiadiffusa: Antihypertensive bis-γ-pyrone polypropionates attenuate angiotensinconverting enzyme. Nat. Prod. Res..

[5] Mayada M.E., Nermeen A.E., Ahmed M.A., Ahmed M.B., Khaled M.D., Sameh S.E., Mostafa M.S., Safwat A.A. (2024). In vivo determination of analgesic and anti-inflammatory activities of isolated compounds from Cleome amblyocarpa and molecular modelling for the top active investigated compounds. RSC Adv..

[6] Mohd K.H., Mohammad F.K., Shahnaaz K., Abdullah G.A., Chromenes M.S. (2020). Phytomolecules with immense therapeutic potential. Plant-derived Bioactives: Chemistry and Mode of Action.

[7] Maddahi M., Asghari S., Pasha G.F. (2023). A facile one-pot green synthesis of novel 2- amino-4 H-chromenes: Antibacterial and antioxidant evaluation. Res. Chem. Intermed..

[8] Triveena M.R., Maha A.E., Eman A.F. (2023). Synthetic coumarin derivatives with anticoagulation and antiplatelet aggregation inhibitory effects. Med. Chem. Res..

[9] Babitha T.D., Raja B., Sathianarayanan S., Baskar V., Muthu T., Ginson G., Maksim R., Gokhan Z., Monica G., Domenico M. (2022). Inhibitory Potential of Chromene Derivatives on Structural and Non-Structural Proteins of Dengue Virus. Viruses.

[10] Hari M., Ehtesham J., Mohammad R., Nasimul H. (2022). Recent advancements in chromone as a privileged scaffold towards the development of small molecules for neurodegenerative therapeutics. RSC Med. Chem..

[11] Ghomashi S., Ghomashi R., Damavandi M.S., Fakhar Z., Mousavi S.Y., Jazi A.S., Gharaghani S., Massah A.R. (2024). Evaluation of antibacterial, cytotoxicity, and apoptosis activity of novel chromene-sulfonamide hybrids synthesized under solvent-free conditions and 3D-QSAR modeling studies. Sci. Rep..

[12] Fouda A.M., Hassan A.H., Eliwa E.M., Ahmed H.E.A., Al-Dies A.M., Omar A.M., Nassar H.S., Halawa A.H., Aljuhani N., El-Agrody A.M. (2020). Targeted potent antimicrobial benzochromene-based analogues: Synthesis, computational studies, and inhibitory effect against 14α-demethylase and DNA gyrase. Bioorganic Chem..

[13] El-Wahab A.H.F.A., Borik R.M., Al-Dies A.M., Fouda A.M., Mohamed H.M., El-Eisawy R.A., Sharaf M.H., Alzahrani A.Y.A., Elhenawy A.A., El-Agrody A.M. (2024). Targeted potent antimicrobial and antitumor oxygen-heterocyclic-based pyran analogues: Synthesis and computational studies. Sci. Rep..

[14] Murali K.V., Rambabu B., Vishnu T., Vani T., Lakshmi S.B., Vijjulatha M. (2024). Antioxidant and antimicrobial activities of 4H-Chromene based indole-pyrimidine hybrids: Synthesis and molecular docking studies. Chem. Biodivers..

[15] Zhao W., Wang B., Liu Y., Fu L., Sheng L., Zhao H., Lu Y., Zhang D. (2020). Design, synthesis, and biological evaluation of novel 4H-chromen-4-one derivatives as antituberculosis agents against multidrug-resistant tuberculosis. Eur. J. Med. Chem..

[16] Surendra B.L., Rajendra P.Y., Srinath N., Richie R.B., Venu S.G., Bontha V.S.L., Rahman M.M., Afzal B.S. (2022). Antitubercular activity assessment of fluorinated chalcones, 2- minopyridine-3-carbonitrile and 2-amino-4H-pyran-3-carbonitrile derivatives: In vitro, molecular docking and in-silico drug likeliness studies. PLoS ONE.

[17] Lei C., Behnaz A., Ning C., Hongwei L., Chunyan Z., Pengfei L., Juan Z. (2025). TiO_2_/ZnWO_3_ with improved photocatalytic performance in the preparation of chromeno [4,3-b]chromenes, as cardiovascular drugs. J. Mol. Struct..

[18] Florian S., Madeleine G., Matthias R., Ion A., Bernhard B., Rainer S., Thomas M. (2019). New naphthopyran analogues of LY290181 as potential tumor vascular-disrupting agents. Eur. J. Med. Chem..

[19] Niloofar J., Ali A., Ehsan S., Mohammad A.L., Fatemeh S., Mohammad M., Bagher A., Aida I., Hojjat R., Gholamreza H. (2025). Synthesis and evaluation of nitrochromene derivatives as potential antileishmanial therapeutics through biological and computational studies. Sci. Rep..

[20] Naya L.R., Elizama S.S., Francisco R.M.F., Ticiana M.A., Edilberto R.S., Ana B.A., Maria J.M., Luzia K.A.M.L., Almeida M.L. (2025). Amburana cearensis (Cumaru) and its active principles as source of anti-Leishmania drugs: Immunomodulatory activity of Coumarin (1,2-Benzopyrone). Biomedicines.

[21] Smith C.W., Bailey J.M., Billingham M.E.J., Chandrasekhar S., Dell C.P., Harvey A.K., Hicks C.A., Kingston A.E., Wishart G.N. (1995). The anti-rheumatic potential of a series of 2,4-disubstituted-4H-naphtho [1,2-b]pyran-3-carbonitriles. Bioorganic Med. Chem. Lett..

[22] Dmitry I.P., Denis S.Z., Viktoriya M.R., Eduard T.O. (2022). Chromone derivatives suppress neuroinflammation and improve mitochondrial function in the sporadic form of Alzheimer’s disease under experimental conditions, Iran. J. Basic Med. Sci..

[23] Zhi X., Qingtai C., Yan Z., Changli L. (2021). Coumarin-based derivatives with potential anti-HIV activity. Fitoterapia.

[24] Arnab M., Satyajit S., Arisha A., Sujisha S.N., Siddhartha S.G., Abu-Taleb K. (2024). Lproline-catalysed synthesis of chromeno [2,3-b]chromene from 4-hydroxy-2Hchromene-2-thione and an anti-proliferative study. Org. Biomol. Chem..

[25] Mahmoud N.M.Y., Usama F., Nabil M.Y. (2023). Synthesis and anticancer activity of novel Chromene derivatives, Chromeno [2,3-d][1,3]Oxazines, and Chromeno [2,3-d] pyrimidines. Med. Chem..

[26] Elgaafary M., Fouda A.M., Mohamed H.M., Hamed A., El-Mawgoud H.K.A., Jin L., Ulrich J., Simmet T., Syrovets T., El-Agrody A.M. (2021). Synthesis of β-enaminonitrile linked 8-methoxy-1H-benzo[f]chromene moieties and analysis of their antitumor mechanisms. Front. Chem..

[27] El Gaafary M., Lehner J., Fouda A.M., Hamed A., Ulrich J., Simmet T., Syrovets T., El-Agrody A.M. (2021). Synthesis and evaluation of antitumor activity of 9-methoxy-1H-benzo[f]- chromene derivatives. Bioorganic Chem..

[28] Viji A., Balachandran V., Babiyana S., Narayana B., Saliyan V.V. (2020). Molecular docking and quantum chemical calculations of 4-methoxy-{2-[3-(4-chlorophenyl)-5-(4-(propane-2-yl) PHENYL)-4, 5-dihydro-1*H*-pyrazol-1-yl]- 1, 3-thiazol-4-yl}phenol. J. Mol. Struct..

[29] Silverstein M., Basseler G.C., Morill G. (1981). Spectrometric Identical Cation of Organic Compound.

[30] Raja B., Balachandran V., Revathi B., Karabacak M., Periandy S. (2015). Spectroscopic (FT-IR, FT-Raman, NMR and UV–Vis), structural, NBO and HOMO–LUMO analysis of 4-chlorobenzonitrile by quantum chemical methods. Spectrochim. Acta Part A Mol. Biomol. Spectrosc..

[31] Krishnakumar V., Surumbarkuzhali N., Muthunatesan S. (2009). Scaled quantum chemical studies on the vibrational spectra of 4-bromo benzonitrile. Spectrochim. Acta A.

[32] Altun A., Golcuk K., Kumru M. (2003). Theoretical and Experimental Studies of Vibrational Spectra of m-Methylaniline. J. Mol. Struct..

[33] Krishnakumar K. (2003). FTIR and FT-Raman spectra and normal coordinate analysis of 4-chlorobenzoic acid. Indian J. Pure Appl. Phys..

[34] Singh N.P., Yadav R.V. (2001). Vibrational spectra and normal coordinate analysis of 2-nitroaniline. Indian J. Phys. B.

[35] Selvamani C., Balachandran V., Karabacak M., Periandy S. (2014). FT-IR, FT-Raman, NMR and UV–visible spectroscopic investigation, first-order hyperpolarizability, HOMO–LUMO, NBO and thermodynamic analysis of 4-fluoro-2-methylaniline using quantum chemical calculations. Spectrochim. Acta Part A Mol. Biomol. Spectrosc..

[36] Dollish F.R., Fateley W.G., Bentley F.F. (1974). Characteristic Raman Frequencies of Organic Compounds.

[37] Shanmugapriya N., Revathi B., Balachandran V. (2021). Spectroscopic (FT-IR, FT-Raman, NMR and UV), HOMO–LUMO, NBO and thermodynamic analysis of 2,4-dimethoxybenzoic acid by quantum chemical methods. Heliyon.

[38] Sivakumar C., Revathi B., Balachandran V., Karabacak M., Murugavel P. (2021). Combined experimental and theoretical investigation on the vibrational, electronic, NBO, HOMO–LUMO, and MEP analyses of 2,3-difluoroaniline. J. Mol. Struct..

[39] Benzon K.B., Balachandran V., Revathi B., Karabacak M. (2015). FT-IR, FT-Raman, NMR and UV–visible spectra, HOMO–LUMO, NBO and thermodynamic investigation of 3-chloro-2-fluoroaniline using DFT methods. Spectrochim. Acta Part A Mol. Biomol. Spectrosc..

[40] Shana Praveen S., Balachandran V., Revathi B., Karabacak M. (2016). FT-IR, FT-Raman, NMR and UV–Vis spectra, NBO, HOMO–LUMO, MEP and thermodynamic studies of 2-methoxybenzoic acid by DFT calculations. J. Mol. Struct..

[41] Al-Tamimi A.-M.S., Mary Y.S., Hassan H.M., Resmi K., El-Emam A.A., Narayana B., Sarojini B. (2018). Sarojini Study on the structure, vibrational analysis and molecular docking of fluorophenyl derivatives using FT-IR and density functional theory computations. J. Mol. Struct..

[42] Viji A., Balachandran V., Babiyana S., Narayana B., Salian V.V. (2020). FT-IR and FT-Raman investigation, quantum chemical studies, molecular docking study and antimicrobial activity studies on novel bioactive drug of 1-(2,4-Dichlorobenzyl)-3-[2-(3-(4-chlorophenyl)-5-(4-(propan-2-yl)phenyl-4,5-dihydro-1H-pyrazol-1-yl]-4-oxo-4,5-dihydro-1,3-thiazol-5(4H)-ylidence]-2,3-dihydro-1H-indol-2-one. J. Mol. Struct..

[43] Lee C., Yang W., Parr R.G. (1988). Development of the Colle-Salvetti Correlation-Energy Formula into a Functional of the Electron Density. Phys. Rev. B.

[44] Yu J., Su N.Q., Yang W. (2022). Describing Chemical Reactivity with Frontier Molecular Orbitalets. JACS Au.

[45] Dennington R., Kerth T., Millam J. (2009). GaussView, Version 5.

[46] Sharma K., Melavanki R., Patil S.S., Kusanur R., Patil N.R., Shelar V.M. (2019). Spectroscopic behavior, FMO, NLO and NBO analysis of two novel Aryl Boronic Acid derivatives: Experimental and Theoretical Insights. J. Mol. Struct..

[47] Soto C.A.T., Costa A.C., Ramos J.M., Vieira L.S., Rost N.C.V., Versiane O., Rangel J.L., Mondragón M.A., Raniero L., Martin A.A. (2013). Surface enhanced Raman scattering, electronic spectrum, natural bond orbital, and mulliken charge distribution in the normal modes of diethyldithiocarbamate copper (II) complex,[Cu (DDTC)_2_]. Spectrochim. Acta A Mol. Biomol. Spectrosc..

[48] Viji A., Revathi B., Balachandran V., Babiyana S., Narayana B., Salian V.V. (2020). Analysis of spectroscopic, quantum chemical calculations, molecular docking, RDG, ELF, anticancer and antimicrobial activity studies on bioactive molecule 2-[3-(4-Chlorophenyl)-5-(4-(propane-2-yl) phenyl-4,5-dihydro-1H-pyrazol-1-yl]-4-(4-methoxyphenyl)-1,3-thiazol. Chem. Data Collect..

[49] Frisch M.J., Trucks G.W., Schlegel H.B., Scuseria G.E., Robb M.A., Cheeseman J.R., Scalmani G., Barone V., Mennucci B., Petersson G.A. (2013). Gaussian 09, Revision D.01.

[50] Morris G.M., Huey R., Lindstrom W., Sanner M.F., Belew R.K., Goodsell D.S., Olson A.J. (2009). AutoDock4 and AutoDockTools4: Automated docking with selective receptor flexibility. J. Comput. Chem..

[51] Abraham M.J., Murtola T., Schulz R., Páll S., Smith J.C., Hess B., Lindahl E. (2015). GROMACS: High performance molecular simulations through multi-level parallelism from laptops to supercomputers. SoftwareX.

[52] Berendsen H.J.C., Van Der Spoel D., Van Drunen R. (1995). GROMACS: A message-passing parallel molecular dynamics implementation. Comput. Phys. Commun..

[53] Hess B., Kutzner C., van der Spoel D., Lindahl E. (2008). GROMACS 4: Algorithms for highly efficient, load-balanced, and scalable molecular simulation. J. Chem. Theory Comput..

[54] Lindahl E., Hess B., van der Spoel D. (2001). GROMACS 3.0: A package for molecular simulation and trajectory analysis. Mol. Model. Annu..

[55] Pronk S., Páll S., Schulz R., Larsson P., Bjelkmar P., Apostolov R., Shirts M.R., Smith J.C., Kasson P.M., Van Der Spoel D. (2013). GROMACS 4.5: A high-throughput and highly parallel open source molecular simulation toolkit. Bioinformatics.

[56] Van Der Spoel D., Lindahl E., Hess B., Groenhof G., Mark A.E., Berendsen H.J. (2005). GROMACS: Fast, flexible, and free. J. Comput. Chem..

[57] Pettersen E.F., Goddard T.D., Huang C.C., Couch G.S., Greenblatt D.M., Meng E.C., Ferrin T.E. (2004). UCSF Chimera—A visualization system for exploratory research and analysis. J. Comput. Chem..

[58] Huang J., Rauscher S., Nawrocki G., Ran T., Feig M., de Groot B.L., Grubmüller H., MacKerell A.D. (2017). CHARMM36m: An improved force field for folded and intrinsically disordered proteins. Nat. Methods.

[59] Vanommeslaeghe K., MacKerell A.D. (2012). Automation of the CHARMM General Force Field (CGenFF) I: Bond perception and atom typing. J. Chem. Inf. Model..

[60] Jorgensen W.L., Chandrasekhar J., Madura J.D., Impey R.W., Klein M.L. (1983). Comparison of simple potential functions for simulating liquid water. J. Chem. Phys..

[61] Bussi G., Donadio D., Parrinello M. (2007). Canonical sampling through velocity rescaling. J. Chem. Phys..

[62] Parrinello M., Aneesur R. (1981). Polymorphic transitions in single crystals: A new molecular dynamics method. J. Appl. Phys..

[63] Darden T., Darrin Y., Lee P. (1993). Particle mesh Ewald: An N log (N) method for Ewald sums in large systems. J. Chem. Phys..

[64] Hess B., Bekker H., Berendsen H.J., Fraaije J.G. (1997). LINCS: A linear constraint solver for molecular simulations. J. Comput. Chem..

[65] Humphrey W., Dalke A., Schulten K. (1996). VMD: Visual molecular dynamics. J. Mol. Graph..

[66] Lu T., Chen F. (2012). Multiwfn: A multifunctional wavefunction analyzer. J. Comput. Chem..

